# Blood Letting as a Diagnostic

**Published:** 1844-04-06

**Authors:** 


BLOOD-LETTING AS A DIAGNOSTIC.

Mr. Barlow has detailed several cases to show that when, in cases of supposed inflammation, there is not any tolerance of loss of blood, but, on the contrary, the patient faints soon when bled in the upright position, the complaint is one of irritation, and not inflammation.—*London Medical Times.*

